# On the feasibility of the Rayleigh cycle for dynamic soaring trajectories

**DOI:** 10.1371/journal.pone.0229746

**Published:** 2020-03-03

**Authors:** David Alexandre, Luca Marino, André Marta, Giorgio Graziani, Renzo Piva

**Affiliations:** 1 Dipartimento di Ingegneria Meccanica e Aerospaziale, Sapienza Universitá di Roma, Rome, Italy; 2 IDMEC, Instituto Superior Técnico, Universidade de Lisboa, Lisbon, Portugal; University of New South Wales, AUSTRALIA

## Abstract

Dynamic soaring is a flight technique used by albatrosses and other birds to cover large distances without the expenditure of energy, which is extracted from the available wind conditions, as brightly perceived five centuries ago by Leonardo da Vinci. Closed dynamic soaring trajectories use spatial variations of wind speed to travel, in principle, indefinitely over a prescribed area. The application of the concept of closed dynamic soaring trajectories to aerial vehicles, such as UAVs, may provide a solution to improve the endurance in certain missions. The main limitation of dynamic soaring is its dependence on the wind characteristics. More than one century ago, Lord Rayleigh proposed a very simple model, based on the repeated crossing of a step wind profile, presently known as Rayleigh cycle, that provides a clear explanation of the physical phenomenon. The present paper studies the feasibility of closed, single-loop, energy-neutral trajectories for a broad set of wind and vehicle conditions. Through the use of trajectory optimization methods, it was possible to see how the shape of the wind profile, the initial flight conditions and the vehicle constraints influence the required wind strength to perform dynamic soaring trajectories and consequently their feasibility. It was possible to conclude that there are optimal values for the initial airspeed and initial height of the vehicle, that minimize the required wind strength. In addition, it was seen how the structural and aerodynamic constraints of the vehicle affect dynamic soaring at high and low airspeeds respectively. Finally, some new trajectories that can be performed in conditions of excess wind are proposed. The purpose is to maximize the time spent aloft and the path length while maintaining the concept of single-loop, energy-neutral trajectories, making them especially useful for aerial vehicles surveillance applications.

## Introduction

The particular flight of sea-birds, especially albatrosses and pelicans, was always admired and repeatedly observed to understand the *secret* of flying without flapping the wings, as usually done by these birds in their flight.

In fact, the way of flying, nowadays referred to as dynamic soaring, is obtained through specific maneuvers, that will be recalled in the following sections. The purpose is to extract energy from the wind to fly, even for long time, without working the wings to obtain the required propulsion. Dynamic soaring requires the presence of a horizontal wind, with a vertical velocity gradient, as usually encountered near the ground or, even more frequently, close to the ocean surface.

Many efforts were spent by scientists, mostly ornithologists and aeronautical engineers [1–3], to reveal the underlying mechanisms of energy harvesting from the wind and many doubts were also raised in the past before arriving at the quite satisfactory understanding reached in the present days.

Among others, Leonardo da Vinci was the first to pay attention to this way of flying and he left many detailed drawings and very interesting descriptions in his famous codexes: namely the Atlantic Codex E and the Codex on the Flight of Birds. He probably captured the essence of this flight with an outstanding intuitive capacity, even though the necessary knowledge of basic physics was not available at his time. For instance, it is worth to mention the description in Codex E (folios 37e, 40r, 41r) very rich of details, as recently reported by Richardson [4]. These findings were practically ignored for many centuries, either for the difficulty to understand ancient Italian and for his typical way of writing mirrored texts.

After almost four centuries, Lord Rayleigh, with comparable ingenuity but with much deeper scientific tools, carefully analyzed the flight of pelicans loitering on the shores and proposed in his famous paper [5] a very interesting model, now well known as the *Rayleigh cycle*. Essentially the model consists of a circular trajectory in a plane inclined downwards to leeward with respect to the horizon in such a way to capture different wind velocity at different heights of the atmospheric boundary layer. To simplify at most the model, Rayleigh considered the very particular step wind profile, presenting an abrupt transition of velocity at a certain level: let us say from zero at ground level to a given wind velocity at the top of the trajectory. This kind of wind profile is not usual in nature but it is an interesting prototype to enhance the essential role of the wind velocity difference for the feasibility of dynamic soaring, as discussed in the following sections.

**Fig 1:**
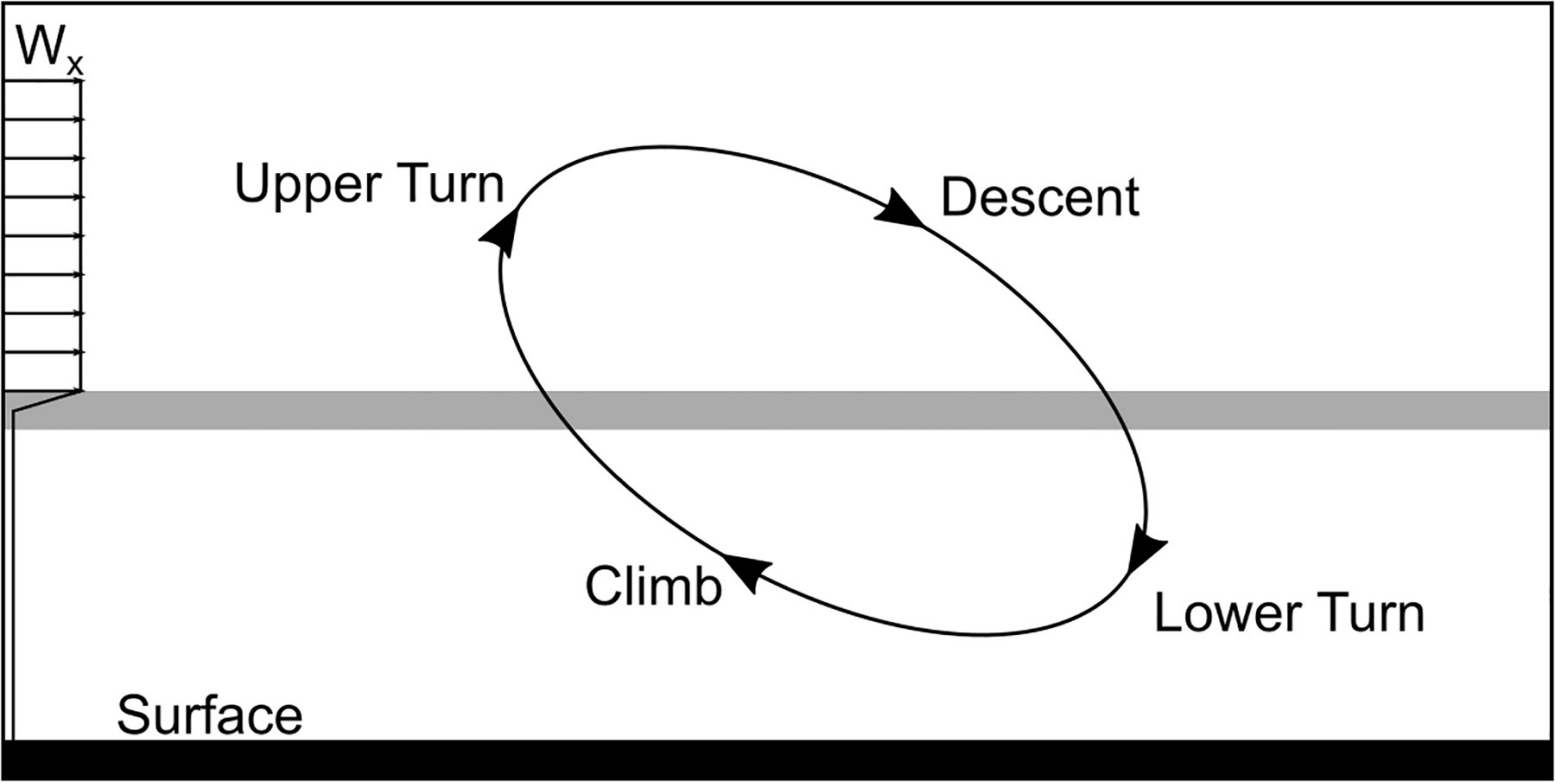
Rayleigh cycle. Schematic representation of the Rayleigh cycle.

To better illustrate Rayleigh’s model, we report in Fig 1 the sketch proposed by Richardson [6] where also a sample of the wind profile appears.

The circular trajectory, following Rayleigh, consists essentially in four different phases in which the bird climbs into the wind, turns at higher level towards leeward direction, descends to ground level along with the wind and finally turns again in the windward direction to reach the initial condition with the same energy, ready to start with a second cycle to continue, possibly, his flight indefinitely. As a consequence of these four phases, a net energy is gained from the wind to balance the dissipation due to aerodynamic drag. At the end of his seminal paper, Rayleigh wrote: “*A priori*, *I should not have supposed the variation of velocity with height to be adequate for the purpose; but if the facts are correct some explanation is badly wanted*”. The aim of the present work is to propose and discuss some results to satisfy the above request.

By the same flying process, based on the energy extraction from the wind, birds can fly for miles and miles, without necessity to flap their wings, during long migrations, as observed by sailors in regions of very strong winds (typically around islands in the southern hemisphere). A certain attention to this subject was given at the beginning mostly by researchers in the field of experimental biology. Among them, Pennycuick [7], through detailed observations and speed measurements, discussed the dynamic soaring technique and estimated the rate of energy consumption. On the basis of his experiences, he suggested that the strength of the measured wind gradient would have been insufficient to maintain airspeed and that most of the energy could be acquired by gliding along waves in a slope lift. A thorough description of dynamic soaring together with its main features, in particular from a biological point of view, is presented in [2]. As one of the precursors for the study of this problem, Cone [8], presented for the first time a very specific and very detailed theoretical explanation of the related phenomena. Dynamic soaring was studied afterwards with many different approaches and the subject became particularly attractive in recent years for the potential applications to unmanned aerial vehicles.

Let us mention here some of the contributions that appeared in the literature starting from the last decades of the past century, that have been of great support for a full comprehension of the subject. Noticeably, Sachs, in a series of papers [9–13], described the mechanism of energy harvesting which allows for dynamic soaring. He also proposed an optimization procedure to evaluate the minimum steepness of the wind gradient required for preserving the energy exchanged with the wind throughout the four phases of the trajectory. In particular, he presented a numerical investigation on minimum wind gradient to obtain energy-neutral trajectories in a linear wind shear [9]. He pointed out also that a significant energy gain is achieved at the end of the windward climb when the turn into the leeward direction is performed. Namely, he claimed that the energy gain in the upper turn could be sufficient to enable dynamic soaring, while the energy increases both in the upwind climb and in the downwind dive are not enough to allow for dynamic soaring [10]. Finally, by discussing again details on the energy transfer mechanisms, he obtained fully equivalent results [11] by performing the energy balance either related to inertial speed or to airspeed [12].

The analysis of neutral energy cycles is presented by Lissamann [14, 15] and an interesting interpretation of the energy extraction mechanism is provided. Bonnin [16] investigated the mechanisms related to energy extraction from the wind by considering a logarithmic wind profile to obtains closed, ∞-shaped, energy-neutral loops.

A study by Richardson [6] closely related to the present one considers a two-layers wind step profile with a very large gradient at the interface. An evaluation of the relevant parameters (mean speed, wind speed, height difference) is examined to demonstrate the feasibility of dynamic soaring. The motion is prescribed along a plane tilted upward into the wind and the trajectory is made of a series of 90° turns connecting upwind climbs and leeward dives repeatedly across the wind shear.

A detailed review of relevant phenomena concerning dynamic soaring is presented in Mir et al. [17], with a particular attention to applications for autonomous vehicles. Recently Kai et al. [18] investigated the effect of dynamic soaring in horizontal wind along an inclined circular path crossing a thin wind shear layer. The flight trajectory is prescribed rather than computed for optimal energy extraction and physical parameters for sustained flight, such as speed increase after a cycle and minimum required wind strength, are discussed. Zhao [19] studied dynamic soaring in terms of a nonlinear optimal control problem by considering the energy balance related to airspeed and different wind profiles. Several target functions are exploited, such as minimum time aloft, maximum altitude gain after each cycle or least required wing gradient slope. In Zhao et al. [20], optimal trajectories related to energy extraction are investigated in several linear wind shear profiles, even with negative gradient (i.e. decreasing wind speed for increasing altitude). Optimal trajectories and dynamic soaring performance for different wind conditions are reported also in [21].

In many of these papers, if not in all, the Rayleigh cycle is mentioned mostly to illustrate the peculiarities of the flight but the attention was then given to migration trajectories, proper to perform long distances, hence typical ocean wind profile were assumed to be more realistic for the simulations. Apart from its theoretical importance, the Rayleigh cycle was, in a way, considered only as an ideal case, not really of interest for applications. A few exceptions are given by Sachs [11], Richardson [6] and by Kai [18] mentioned before.

Purpose of the present paper is the study of feasible closed neutral trajectories able to represent at best the Rayleigh cycle for dynamic soaring in the presence of an approximated step wind profile. By using an optimization algorithm, several solutions are obtained, which minimize the jump of the wind velocity across the layer profile, to verify the feasibility of such closed trajectories in those extreme wind conditions. Considering also several interesting applications for unmanned vehicles, namely for any kind of surveillance covering a certain physical region, different objective functions have been adopted, such as maximum endurance or maximum length of the trajectory. Beforehand, a description of the governing equations and a summary of energy exchange mechanisms in the four phases of the Rayleigh cycle are reported to better introduce the discussion of the simulation results.

## Vehicle and wind models

In order to analyze the Rayleigh cycle, the mathematical model of the flight dynamics has to be defined. The soaring vehicle is considered as a point mass subjected to aerodynamic and gravitational forces in the presence of a wind ***W***.

An inertial Earth fixed reference frame $O(e_{x}^{E},e_{y}^{E},e_{z}^{E})$ and a Flight path frame $O^{\prime }\left(e_{x}^{F},e_{y}^{F},e_{z}^{F}\right)$ moving with the body are introduced to express in a useful form the equation of motion. We assume the Earth axes *x*^*E*^, *y*^*E*^ directed toward the geographic North and East, respectively, while the *z*—axis is in the downward direction.

The velocity in the Earth frame, hereafter named ground velocity ***V***_*G*_, is related to the wind velocity ***W***, through the airspeed ***V***_*A*_ by

$$\begin{array}{c} V_{G}=V_{A}+W. \end{array}$$ (1)

The Flight path frame is inclined with respect to the Earth frame with flight path angle *γ* and rotated with heading angle *ψ* that point the *x*^*F*^ in the airspeed ***V***_*A*_ direction. A wind frame $O^{\prime }\left(e_{x}^{W},e_{y}^{W},e_{z}^{W}\right)$ is also introduced to take into account a possible bank angle *ϕ* through a rotation around the *x*^*F*^ = *x*^*W*^.

**Fig 2:**
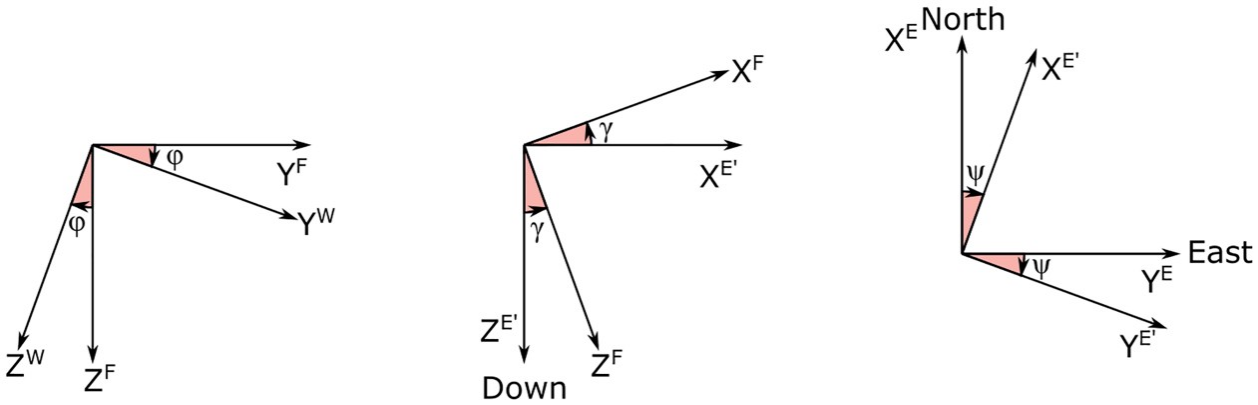
Relation between frames. Sketch of the three rotations ***T***_***x***_(*ϕ*), ***T***_***y***_(*γ*), ***T***_***z***_(*ψ*), that define the Wind (W), Path (F) and Earth frames (E).

Fig 2 shows three simple sketches aiming at clarifying the reader in the comprehension of the relative frame attitude and defining the heading angle *ψ*, the flight path angle *γ* and the bank angle *ϕ*. The change from the Earth reference frame to the Wind frame can be obtained by the application of three successive rotations, as represented by the rotation matrices:

$$\begin{array}{c} T_{x}\left(\phi \right)=[\begin{array}{ccc} 1 & 0 & 0\\ 0 & \;\text{cos}\;\phi & \;\text{sin}\;\phi \\ 0 & -\;\text{sin}\;\phi & \;\text{cos}\;\phi \end{array}],T_{y}\left(\gamma \right)=[\begin{array}{ccc} \;\text{cos}\;\gamma & 0 & -\;\text{sin}\;\gamma \\ 0 & 1 & 0\\ \;\text{sin}\;\gamma & 0 & \;\text{cos}\;\gamma \end{array}],T_{z}\left(\psi \right)=[\begin{array}{ccc} \;\text{cos}\;\psi & \;\text{sin}\;\psi & 0\\ -\;\text{sin}\;\psi & \;\text{cos}\;\psi & 0\\ 0 & 0 & 1 \end{array}], \end{array}$$ (2)

leading to

$$\begin{array}{c} V^{W}=T_{x}T_{y}T_{z}V^{E}, \end{array}$$ (3)

where the superscripts *W* and *E* refer to the velocity components in the Wind and Earth frames, respectively.

Without loss of generality, we assumed the wind direction in the x-axis $e_{x}^{E}$ of the Earth reference frame, i.e., $W=W_{x}^{E}e_{x}^{E}$. As a consequence, the gravitational force can be written in the Earth frame as

$$\begin{array}{c} F_{G}=mge_{z}^{E}, \end{array}$$ (4)

where *m* is the vehicle mass and *g* is the gravitational acceleration, and the aerodynamic force in the wind frame is

$$\begin{array}{c} F_{A}=-De_{x}^{W}-Le_{z}^{W}. \end{array}$$ (5)

The lift *L* and drag *D* can be expressed, as usual, by using the vehicle’s airspeed *V*_*A*_, the air density *ρ*, the wing planform area *S*, the lift coefficient *C*_*L*_ and the drag coefficient *C*_*D*_, yielding

$$\begin{array}{c} L=\frac{1}{2}\rho SV_{A}^{2}C_{L} \end{array}$$ (6)

and

$$\begin{array}{c} D=\frac{1}{2}\rho SV_{A}^{2}C_{D}. \end{array}$$ (7)

The drag coefficient *C*_*D*_ is related to the lift coefficient *C*_*L*_ by the commonly adopted polar equation,

$$\begin{array}{c} C_{D}=C_{D_{0}}+KC_{L}^{2}, \end{array}$$ (8)

where $C_{D_{0}}$ is the zero lift drag coefficient and *K* the coefficient of the lift dependent drag.

Many authors pointed out that the equations of motion can be written in Earth fixed or in the Flight path frame [16, 17]. This issue is particularly important when the energy analysis is carried out to explain the different contributions occurring in the phases of the trajectory. Very recently, Sachs [12] discussed thoroughly the different points of view, showing a substantial equivalence between the two possible approaches, in so clarifying a long standing debate.

For the sake of clarity, here both sets of equations are reported, anticipating that the energy analysis will be considered in the Earth fixed frame.

In the Flight path frame, it is convenient to take the set of equations of motion from Zhao [19] where a different definition of the path angle *γ* is considered

$${\dot{V}}_{A}=-\frac{1}{m}D-g\;\text{sin}\;\gamma -\;\text{cos}\;\gamma \;\text{cos}\;\psi {\dot{W}}_{x}^{E},$$ (9a)

$$V_{A}\;\text{cos}\;\gamma \dot{\psi }=\frac{1}{m}L\;\text{sin}\;\phi +\;\text{sin}\;\psi {\dot{W}}_{x}^{E},$$ (9b)

$$V_{A}\dot{\gamma }=\frac{1}{m}L\;\text{cos}\;\phi -g\;\text{cos}\;\gamma +\;\text{sin}\;\gamma \;\text{cos}\;\psi {\dot{W}}_{x}^{E},$$ (9c)

$$\dot{x}=V_{A}\;\text{cos}\;\gamma \;\text{cos}\;\psi +W_{x}^{E},$$ (9d)

$$\dot{y}=V_{A}\;\text{cos}\;\gamma \;\text{sin}\;\psi ,$$ (9e)

$$\dot{z}=-V_{A}\;\text{sin}\;\gamma ,$$ (9f)

where Eqs (9a)–(9c) represent Newton’s second law and describe the point mass dynamics, whereas Eqs (9d)–(9f) represent the kinematics, in particular the vehicle linear velocity (translation).

In contrast, the corresponding set of dynamic equations in the Earth frame is given by

$$m\ddot{x}=-D\;\text{cos}\;\gamma \;\text{cos}\;\psi -L\left(\;\text{cos}\;\phi \;\text{sin}\;\gamma \;\text{cos}\;\psi +\;\text{sin}\;\phi \;\text{sin}\;\psi \right),$$ (10a)

$$m\ddot{y}=-D\;\text{cos}\;\gamma \;\text{sin}\;\psi -L\left(\;\text{cos}\;\phi \;\text{sin}\;\gamma \;\text{sin}\;\psi -\;\text{sin}\;\phi \;\text{cos}\;\psi \right),$$ (10b)

$$m\ddot{z}=D\;\text{sin}\;\gamma -L\;\text{cos}\;\phi \;\text{cos}\;\gamma +mg.$$ (10c)

The analysis of the motion is completed by considering the related energy exchanges. In particular, the rate of change of mechanical energy, related to inertial velocity ***V***_***G***_, is equal to the power of non-conservative forces (***F***_*NC*_),

$$\begin{array}{c} \frac{dE_{m}}{dt}=\sum \left(F_{NC}\cdot V_{G}\right). \end{array}$$ (11)

Since the non-conservative forces present in the model are lift ***L*** and drag ***D*** only, it is possible to split the rate of change of mechanical energy into two contributions

$$\begin{array}{c} \frac{dE_{m}}{dt}=L\cdot V_{G}+D\cdot V_{G}. \end{array}$$ (12)

By taking into account Eq (1) and the orthogonality between ***L*** and ***V***_*A*_, Eq (12) becomes

$$\begin{array}{c} \frac{dE_{m}}{dt}=L\cdot W+D\cdot V_{A}+D\cdot W. \end{array}$$ (13)

Since $W=W_{x}^{E}e_{x}^{E}$ we obtain

$$\begin{array}{c} \frac{dE_{m}}{dt}=-LW_{x}^{E}\left(\;\text{cos}\;\phi \;\text{sin}\;\gamma \;\text{cos}\;\psi +\;\text{sin}\;\phi \;\text{sin}\;\psi \right)-D\left(V_{A}+W_{x}^{E}\;\text{cos}\;\gamma \;\text{cos}\;\psi \right). \end{array}$$ (14)

Eq (14) deserves some comments. In the absence of wind, $W_{x}^{E}=0$, there is only the negative contribution of *DV*_*A*_ and thus the mechanical energy is strictly a decreasing quantity [16]. When the wind is present, the possibility to have an energy increase depends on the relative contributions given by each term. The values assumed by the each term will be reported later in a section devoted to the detailed analysis of each phase of the soaring trajectories. As shown by (14), the lift contribution is function of both the bank angle *ϕ*, the heading angle *ψ* and the flight path angle *γ*, while the drag term depends only on *ψ* and *γ*. So the possibility of extracting energy by the wind is related to the characteristics of the flight and to the aerodynamic properties of the flying vehicle.

**Fig 3:**
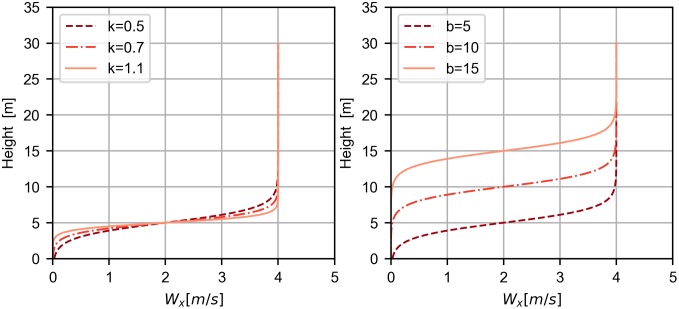
Step wind profiles. Effect of changing parameters k and b in the shape of the step wind profile (here *A* = 4).

Different wind models have been considered by many authors to study the dynamic soaring phenomenon. In particular linear, logarithmic or power law wind profiles have been analyzed. Here the interest is focused on the Rayleigh cycle hence on a step wind model that concentrates in a thin layer the variation of wind velocity. In this case, the wind can be modeled by the following function

$$\begin{array}{c} W=\frac{A}{2}\left(\text{tanh}\left(k\left(h-b\right)\right)+1\right)e_{x}^{E}, \end{array}$$ (15)

where *A* is the maximum wind speed, *h* is the altitude (*h* = −*z*), *k* and *b* are parameters controlling the steepness of the gradient and the transition height where half of the step is reached, respectively. By changing *k* and *b*, in particular, we will assess the feasibility and the characteristics of the resulting trajectories. The studied wind profiles are shown in Fig 3.

## Phases of Rayleigh cycle

The energy-harvesting mechanism associated with a Rayleigh cycle is here presented to give a better comprehension of the results. The goal of an energy-neutral trajectory, by definition, is to gain enough energy from the wind to balance the losses associated to the dissipative phenomena and, in order to achieve a thorough understanding of the energy process, each phase of the trajectory has a peculiar behavior to be analyzed. With reference to Fig 1, the trajectory can be decomposed into four phases: climb, upper turn, descent and lower turn. A clockwise motion is assumed.

### Climb

During the climbing phase, altitude is gained in exchange for a decreasing airspeed. Because the motion direction is opposite to the wind, the ground speed is always lower than the airspeed. In addition, the flight path angle is positive (*γ* > 0), the heading angle lays between $\frac{\pi }{2}$ and $\frac{3\pi }{2}$ since the motion is in the southward direction, the bank angle is positive (*ϕ* ≥ 0).

**Fig 4:**
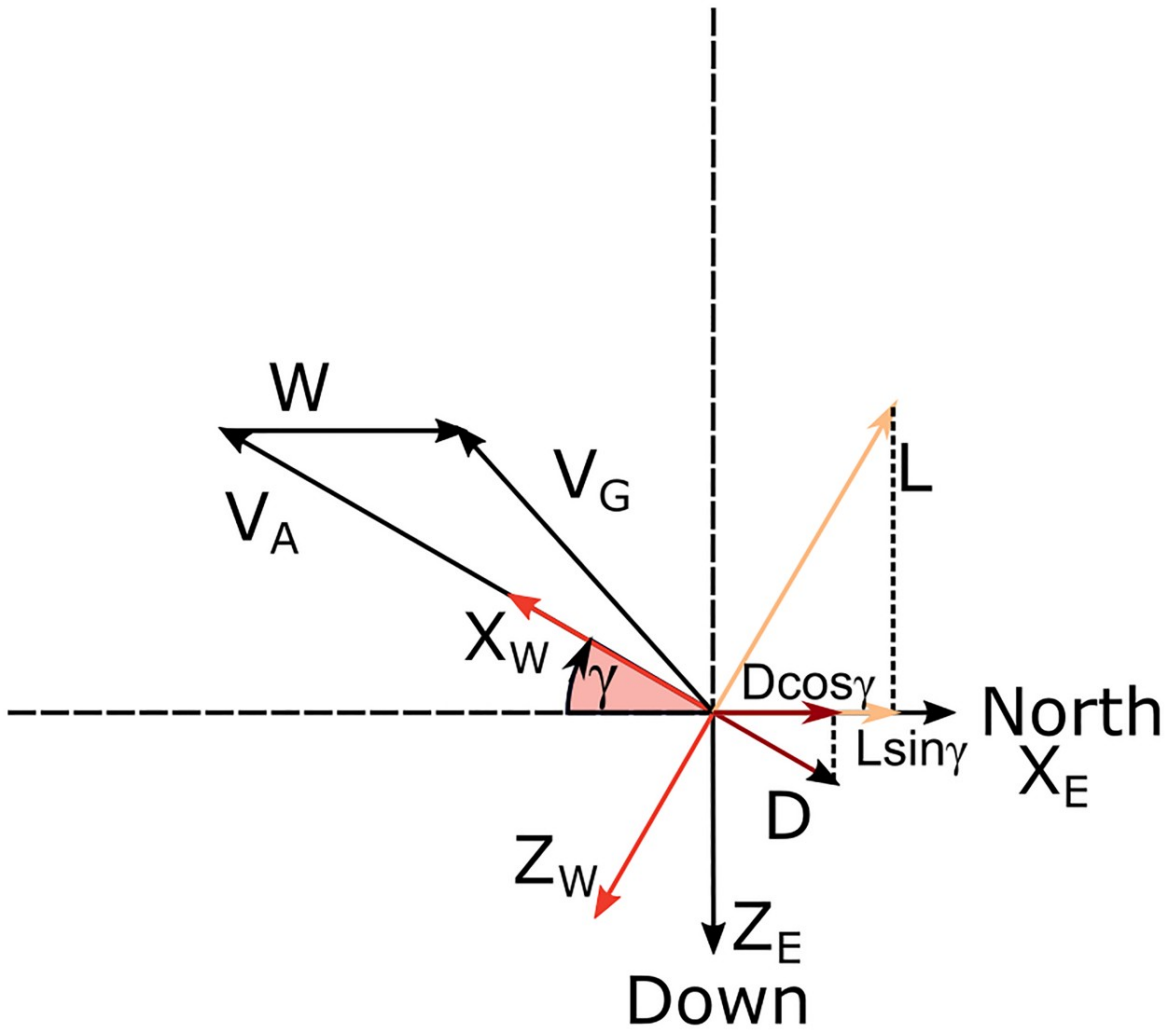
Sketch for the climbing phase. Velocity vectors and forces in the vertical plane for bank angle *ϕ* = 0 and southward heading *ψ* = *π*.

The lift gives a positive contribution to the energy budget, also explained in Fig 4 where the projection of **L** along the wind velocity **W** is positive. Moreover, this contribution is increasing with time as a result of the corresponding increasing wind speed strength.

To better understand how the lift can contribute positively to the overall energy of the system, it is useful to consider a simplified case in which the climb occurs with bank angle *ϕ* = 0 and motion pointing directly southward (*ψ* = *π*). Without loss of generality, following these assumptions Eq (14) becomes

$$\begin{array}{c} \frac{dE_{m}}{dt}=LW_{x}^{E}\;\text{sin}\;\gamma +DW_{x}^{E}\;\text{cos}\;\gamma -DV_{A}, \end{array}$$ (16)

and for a positive variation of the mechanical energy

$$\begin{array}{c} \frac{L}{D}\frac{W_{x}^{E}}{V_{A}}\;\text{sin}\;\gamma +\frac{W_{x}^{E}}{V_{A}}\;\text{cos}\;\gamma -1>0, \end{array}$$ (17)

which means that during the climb the energy gain depends mainly on the wind strength and on the L/D ratio.

We observe that even if the term $DW_{x}^{E}\;\text{cos}\;\gamma$ in Eq (16) is positive, its contribution is smaller than *DV*_*A*_.

### Upper turn

The next phase of the flight is the upper turn. During this phase, the positive bank angle (*ϕ* > 0) allows for a 180° turn to the right, letting the change of flight direction from southward to northward.

In this phase, the maximum altitude and minimum airspeed are reached and the leeward descent is eventually started. In addition, the flight path angle *γ* goes from positive to negative in preparation for the descent phase.

**Fig 5:**
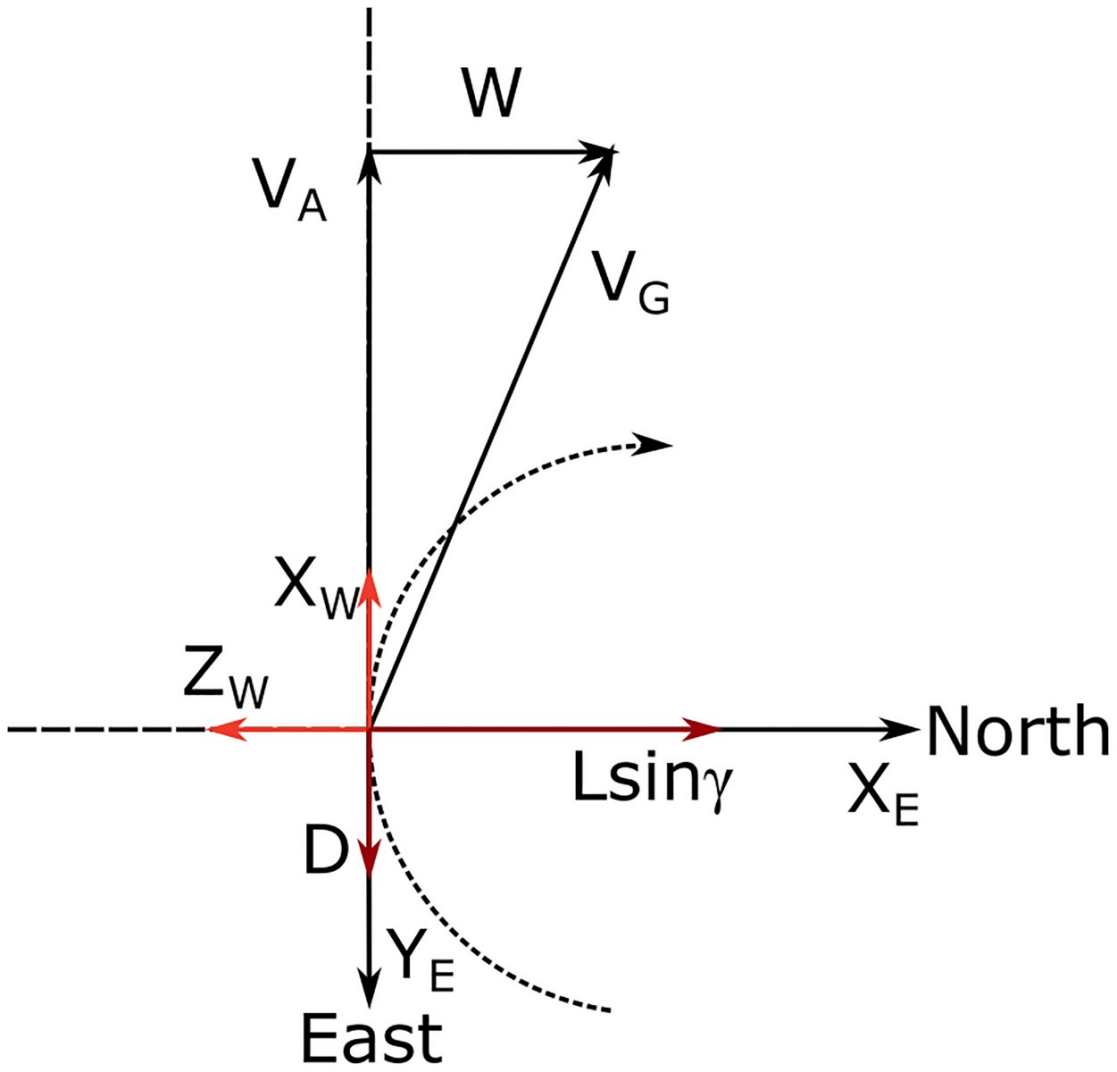
Sketch for the upper turn phase. Velocity vectors and forces in the horizontal plane for the middle of the upper turn (*ψ* = 3*π*/2) and for level flight (*γ* = 0).

Fig 5 presents a sketch of the forces acting in this phase for the simplified case where it is assumed the turn to occur in level flight (*γ* = 0). In this condition, Eq (14) becomes

$$\begin{array}{c} \frac{dE_{m}}{dt}=-LW_{x}^{E}\;\text{sin}\;\phi \;\text{sin}\;\psi -DV_{A}-DW_{x}^{E}\;\text{cos}\;\psi . \end{array}$$ (18)

During a turn from *ψ* = *π* to *ψ* = 2*π*, sin *ψ* < 0 and sin *ϕ* > 0, resulting in a positive contribution from the lift throughout the turn.

### Descent

The descent phase is characterized by a loss of altitude in exchange for a gain in airspeed. The ground speed is always higher than the airspeed due to the tail wind.

For the case of the Rayleigh cycle here considered, the bank angle is still positive (*ϕ* > 0) while the flight path angle is negative (*γ* < 0), with a northward pointing heading ($-\frac{\pi }{2}<\psi <\frac{\pi }{2}$).

If the simplified case of descent without banking (*ϕ* = 0) and facing directly north (*ψ* = 0) is considered, then Eq (14) becomes

$$\begin{array}{c} \frac{dE_{m}}{dt}=-LW_{x}^{E}\;\text{sin}\;\gamma -DV_{A}-DW_{x}^{E}\;\text{cos}\;\gamma \end{array}$$ (19)

and, to insure a positive rate for the mechanical energy, the following condition must be respected

$$\begin{array}{c} -\frac{L}{D}\frac{W_{x}^{E}}{V_{A}}\;\text{sin}\;\gamma -\frac{W_{x}^{E}}{V_{A}}\;\text{cos}\;\gamma -1>0. \end{array}$$ (20)

**Fig 6:**
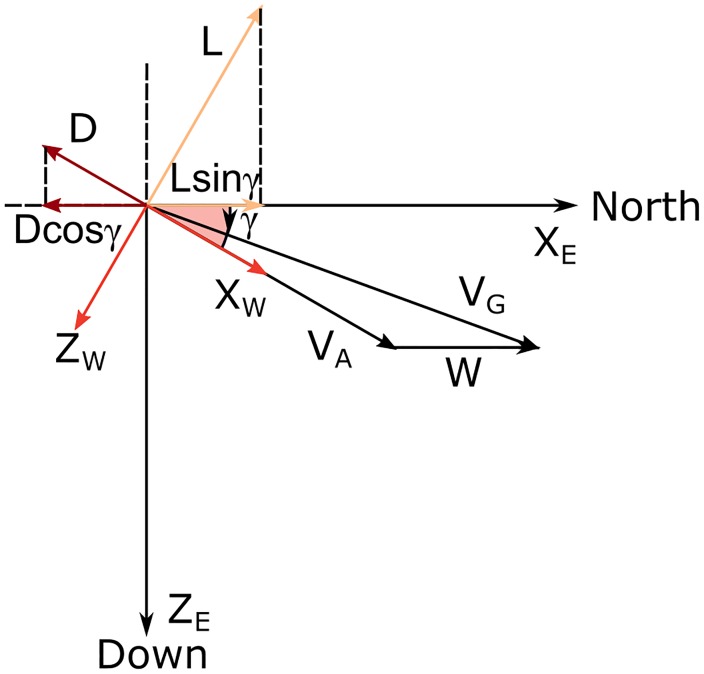
Sketch for the descent phase. Velocity vectors and forces during the descent phase with no bank (*ϕ* = 0) and heading north (*ψ* = 0).

It is then clear that the lift contribution is still positive and, just as in the climb, it depends both on wind strength and L/D ratio. Fig 6 presents the schematic representation of the parameters involved in the descent.

### Lower turn

Finally, the last phase of the flight is the lower turn that will recover the initial condition. The phase is characterized as the part of the flight where energy losses occur.

While the bank continues to be positive (*ϕ* > 0), the flight path angle goes from negative to positive and the heading changes from northward to southward in preparation for another loop.

The negative contribution of the lift during this phase is better understood when considering the simplified case in which it is assumed that the turn occurs with flight path angle equal to zero (*γ* = 0), simplifying Eq (14) to

$$\begin{array}{c} \frac{dE_{m}}{dt}=-LW_{x}^{E}\;\text{sin}\;\phi \;\text{sin}\;\psi -DV_{A}-DW_{x}^{E}\;\text{cos}\;\psi . \end{array}$$

**Fig 7:**
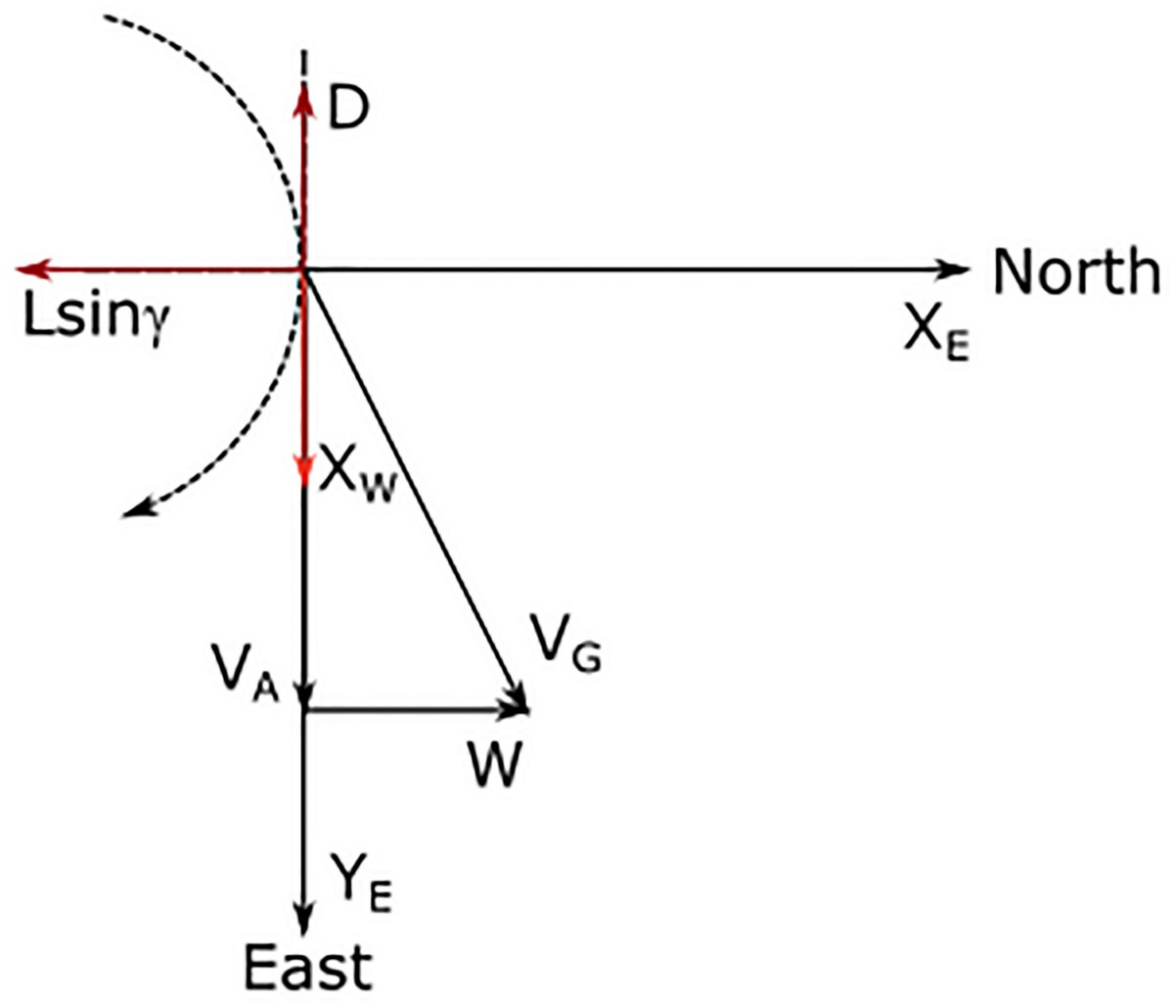
Sketch for the lower turn. Velocity vectors and forces in the horizontal plane during the lower turn for *γ* = 0 and *ψ* = *π*/2.

Looking at this equation it is possible to verify that, when wind is present, the lift contributes negatively, since sin *ψ* > 0 in a turn from 2*π* to 3*π* (which recovers the initial condition). Fig 7 presents the schematic representation of the present phase, where *F* represents the component of the lift that acts as an additional drag contribution. In fact *L* sin *ϕ* is parallel but opposite to *W* and *D* is parallel but opposite to *V*_*A*_ so both contributions are negative and reduce the energy.

## Solution procedure

### Problem statement

The study of dynamic soaring trajectories can be seen as an optimization problem with a proper objective function [22]. Considering the equations of motion (9) in the flight path frame, it is possible to assume as the state vector

$$\begin{array}{c} x=[\begin{array}{cccccc} x & y & z & V_{A} & \psi & \gamma \end{array}], \end{array}$$ (21)

and

$$\begin{array}{c} u=[\begin{array}{cc} C_{L} & \phi \end{array}]. \end{array}$$ (22)

as the control vector of the trajectory optimization problem.

To obtain the energy-neutral Rayleigh cycle from the equations of motion, it is usual [10] and of major interest to minimize the required wind strength *A* for dynamic soaring, following (15).

We notice that, by forcing the final heading angle *ψ* to be 2*π* greater than the initial one, the single loop trajectory is assured. Moreover, Table 1 presents the lower and upper limits of the state variables, as well as the boundary conditions for the problem in the form of the initial and final values for the state vector.

**Table 1:** Bounds, initial and final values for the state variables. Since the trajectories of interest are closed and energy-neutral, in general, ***x***_**0**_ = ***x***_***F***_.

Variable	*x*[m]	*y*[m]	*h*[m]	*V*_*A*_[m/s]	*ψ*[rad]	*γ*[rad]
Lower limit (***x***_***min***_)	−100	−100	1.5	0	−inf	$\frac{-\pi }{3}$
Upper limit(***x***_***max***_)	100	100	100	50	inf	$\frac{\pi }{3}$
Initial Value (***x***_**0**_)	0	0	1.5	20	$\frac{\pi }{2}$	0
Final Value (***x***_***f***_)	0	0	1.5	20	$\frac{5\pi }{2}$	0

As shown in Table 2, the control variables are also bounded between a lower and a upper value, on the basis of reasonable aerodynamic performance.

**Table 2:** Acceptable range of the control variables.

Variable	*C*_*L*_[-]	*ϕ*[rad]
Lower limit (***u***_***min***_)	0	$-\frac{\pi }{3}$
Upper limit (***u***_***max***_)	1.5	$\frac{\pi }{3}$

In addition to the bounds of the state and control variables, during the trajectory a maximum load factor *n*, representing a structural limitation, is also prescribed as

$$\begin{array}{c} n=\frac{L}{W}=\frac{0.5\rho SV_{A}^{2}C_{L}}{mg}\leq n_{max}. \end{array}$$ (23)

In the present case, we chose *n*_*max*_ = 3 as a quite conservative value.

Purpose of the optimization procedure is to minimize the wind strength, resulting for the case of a step wind profile Eq (15) in an objective function

$$\begin{array}{c} \text{min}\;A. \end{array}$$ (24)

The vehicle parameters adopted in the present analysis, summarized in Table 3, match those found in Sachs [10].

**Table 3:** Vehicle parameters adopted in the present analysis [10].

*m* [*kg*]	*S* [*m*^2^]	$C_{D_{0}}$	*K*
8.5	0.6	0.033	0.019

### Computational method

The trajectory optimization problem just stated can be solved using different techniques. Following the work of several authors [16, 19, 23] a direct method will be used. The method has essentially two phases: a transcription phase that converts the problem into a non linear program (NLP); and a solving phase where a NLP solver applies an optimization algorithm to find the solution.

To transcribe the problem, the continuous trajectory is discretized into a finite set of points in time, called collocation points, each with a specific value for the state and control variables. So, if the vehicle’s trajectory is discretized into N points in a time interval [0, *t*_*f*_], there are N time unknowns,

$$\begin{array}{c} t=t_{0},\ldots ,t_{k},\ldots ,t_{N}, \end{array}$$ (25)

6N state unknowns, the *x*, *y* and *z* position coordinates of the vehicle, the airspeed *V*_*A*_ and the heading and flight path angles, *ψ* and *γ*, for each collocation point k,

$$\begin{array}{c} x=x_{0}^{i},\ldots ,x_{k}^{i},\ldots ,x_{N}^{i},i=1,\ldots \;6, \end{array}$$ (26)

and 2N control unknowns, the lift coefficient *C*_*L*_ and bank angle *ϕ*

$$\begin{array}{c} u=u_{0}^{j},\ldots ,u_{k}^{j},\ldots ,u_{N}^{j},j=1,2, \end{array}$$ (27)

resulting in a total of 8N unknowns. This set of unknowns are the variables for the optimization procedure Note that after determining the initial and final time of the trajectory, all time unknowns can be calculated using the spacing between the collocation points [22, 23].

The solution of the discretized differential equations has been obtained by means of trapezoidal rule integration. For a generic differential equation,

$$\begin{array}{c} \dot{x^{i}}=f^{i}\left(x\left(T\right),u\left(T\right)\right), \end{array}$$ (28)

the use of the trapezoidal rule establishes a relationship between two consecutive collocation points [23],

$$\begin{array}{c} \left(x_{k+1}^{i}-x_{k}^{i}\right)-\frac{1}{2}\left(f_{k}^{i}+f_{k+1}^{i}\right)\left(t_{k+1}-t_{k}\right)=0,k=1,\ldots ,N-1 \end{array}$$ (29)

For the 6 differential equations for the vehicle dynamics, a total of 6(N-1) equality constraints are related to the system dynamics.

The NLP obtained from the transcription can be solved using an interior point method, which converts the constrained NLP into an unconstrained optimization method and then proceeds to use a Newton method to solve the problem [24]. Interior point methods are specially designed to tackle this kind of problems, being robust and well documented methods.

In the developed work, the transcription was made using the Imperial College London Optimal Control Software (ICLOCS2) [25] and the NLP solver chosen was Interior Point Optimizer (IPOPT) [26].

### Rayleigh cycle solution

As a first result of our analysis we present the trajectory and a detailed description of the main variables involved in the model. The optimal trajectory corresponds to a step profile where the steepness and transition height are *k* = 0.5 and *b* = 0.5, respectively.

**Fig 8:**
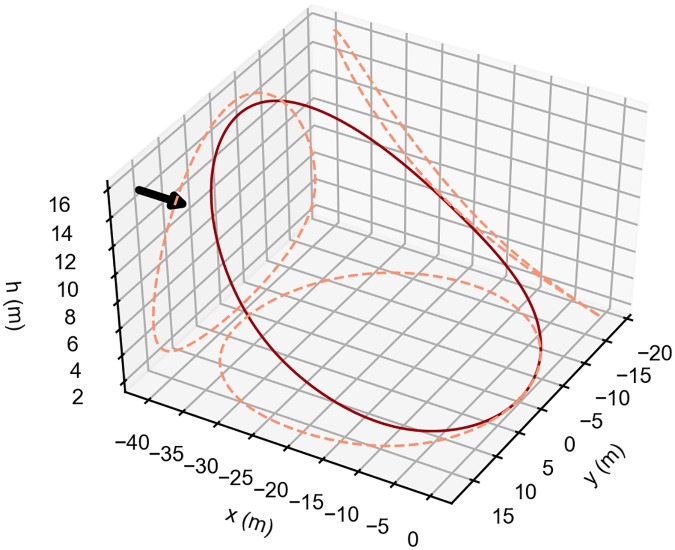
Rayleigh cycle optimal trajectory. Optimal single-loop, energy-neutral trajectory that minimizes the wind strength required in a step wind profile. Arrow indicates the wind direction. The dashed lines represent the trajectory projections on each of the three coordinates planes. The vehicle travels in the clockwise direction.

Fig 8 shows the shape of the Rayleigh cycle as well as the projection of the trajectory on the three Cartesian planes.

**Fig 9:**
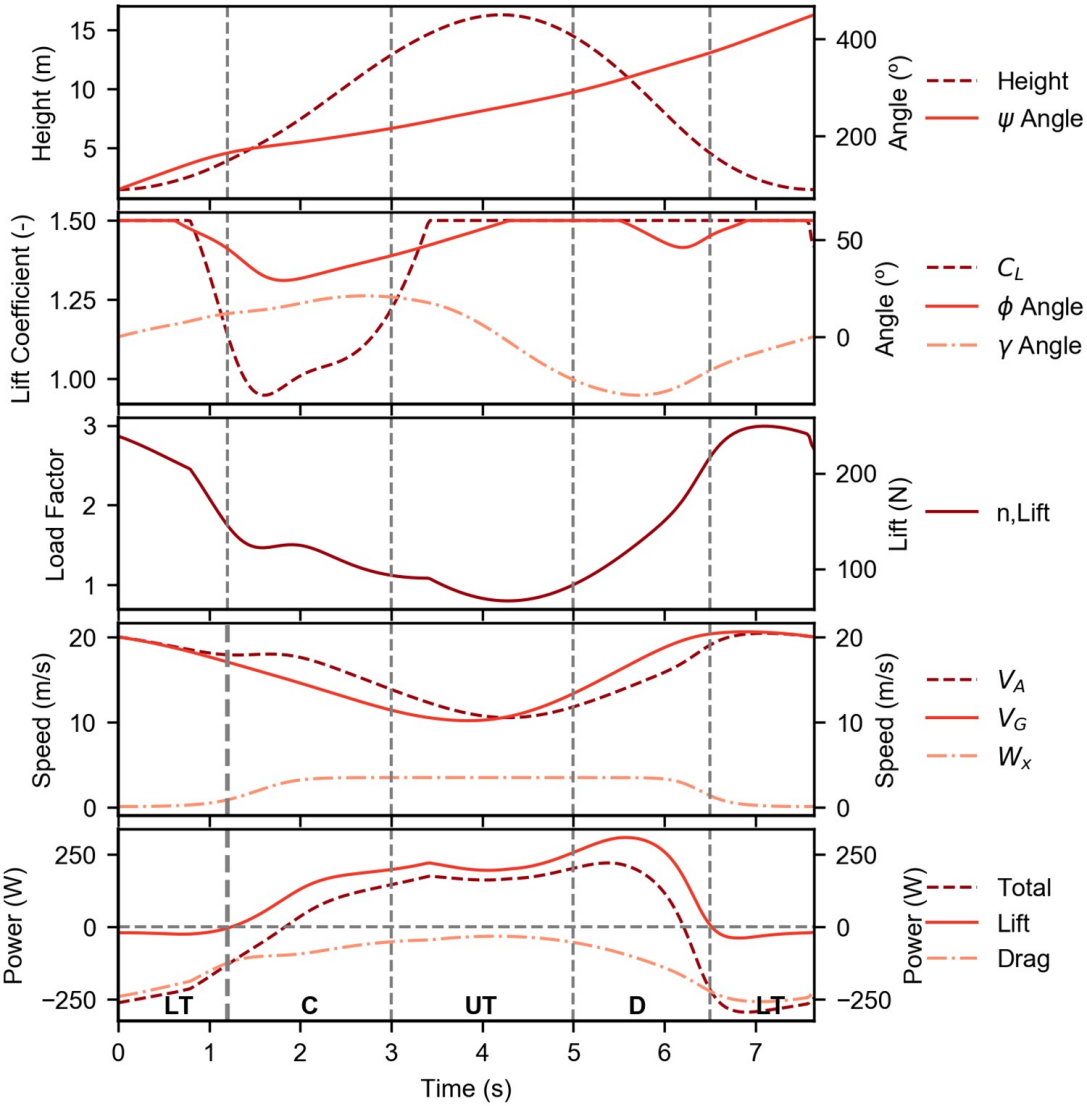
Evolution of the Rayleigh cycle. Detailed time evolution of the main parameters of the optimal trajectory that minimizes the wind strength required for a step wind profile (*k* = 0.5, *b* = 0.5). The first panel presents the altitude and heading angle. The second panel presents the control variables and the flight path angle. The third panel presents the lift and load factor (the lines are coincident). The fourth panel presents the ground speed, airspeed and wind speed. Finally, the last panel presents the lift and drag contributions for the variation of the mechanical energy, as well as the overall variation of the energy (LT—Lower Turn, C—Climb, UT—Upper Turn, D—Descent).

Fig 9 illustrates the detailed evolution of the main parameters for the complete cycle. It is possible to appreciate, in the lower panel, the four phases previously described. In particular, it is possible to observe a positive contribution of the lift *L* to the total energy rate for almost all the climb, the upper turn and the descent phases. A negative value is noticed in the lower turn phase. In the same panel, it is also shown the drag *D* effect in the different part of the trajectory as well as the total energy rate evolution.

Furthermore, the control variables *C*_*L*_ and *ϕ* display an interesting behavior, in particular the lift coefficient changes almost only in the climb phase whereas in the remaining phases reaches the maximum value. When *C*_*L*_ = *C*_*Lmax*_, the changes in lift are given by the changes in airspeed *V*_*A*_. The optimization results in the maximum lift possible in the phases where the energy exchange from the wind is favorable. The bank angle *ϕ* follows quite closely the behavior of the lift coefficient *C*_*L*_, even if it is less constant.

## Feasibility analysis

Purpose of the present section is to find how the wind profile, the initial conditions and vehicle constraints affect dynamic soaring trajectories and their feasibility.

To compare the trajectories corresponding to the different parameters of the wind profiles, we introduce the quantity

$$\begin{array}{c} \Delta W=W\left(h_{max}\right)-W\left(h_{min}\right), \end{array}$$ (30)

where *h*_*max*_ and *h*_*min*_ are the highest and lowest altitudes achieved during the trajectory, respectively. Since the wind gradient is always positive, Δ*W* compares the maximum and minimum wind speeds experienced by the vehicle.

We stress that the focus of the paper is to study the feasibility of the Rayleigh cycle, so trajectories that require too high wind strengths can be considered as inefficient.

The present feasibility study considers, as described below, four parameters: wind profile, vehicle initial conditions (height and airspeed), vehicle constraints (maximum lift coefficient and maximum load factor), and excess wind conditions.

### Effect of the wind profile

Table 4 summarizes the main characteristics of the closed, energy-neutral trajectories that minimize the required wind strength *A*, obtained for the different wind profiles.

**Table 4:** Comparison between dynamic soaring trajectories for different wind profiles, for initial airspeed of 20m/s and initial height of 1.5m.

Wind Profile	*t*_*s*_ [s]	*h*_*max*_ [m]	*l* [m]	Δ*W* [m/s]
Step 1 (k = 0.5, b = 5)	7.64	16.26	119.29	3.40
Step 2 (k = 0.5, b = 10)	7.85	16.00	117.36	3.86
Step 3 (k = 0.5, b = 15)	9.05	18.28	119.00	6.46
Step 4 (k = 0.7, b = 5)	7.59	16.31	118.62	3.31
Step 5 (k = 1.1, b = 5)	7.56	16.27	118.28	3.23

Looking at Table 4, it is possible to observe that the least efficient wind profile is the step wind profile with transition height equal to 15 meters, which requires almost a double wind strength when compared with the other wind profiles. In contrast, all other wind profiles show a comparable behavior for the considered parameters. It is also possible to notice a slight decrease in the required wind strength for increasing steepness *k* of the step profile.

A comparison with the logarithmic wind profile, usually adopted by most of the authors, is considered. In this case, the model is given by

$$\begin{array}{c} W=W_{ref}\frac{ln(h/h_{0})}{ln(h_{ref}/h_{0})}e_{x}^{E}, \end{array}$$ (31)

where *h*_*ref*_ it is a reference altitude, *h*_0_ is the wind profile starting altitude and *W*_*ref*_ is the wind speed at the reference altitude. For this wind profile Eq (24) is replaced by min *W*_*ref*_. We obtain *t*_*s*_ = 11.57*s*, *h*_*max*_ = 14.82*m*, *l* = 167.29*m* and Δ*W* = 5.16*m*/*s*, hence this profile requires a higher wind strength than the more efficient step wind profiles, even though it has a lower requirement with respect to the step 3 profile.

Table 4 also shows that, in general, while the time and length of the trajectory display small variations, much larger values are obtained for the logarithmic profile.

**Fig 10:**
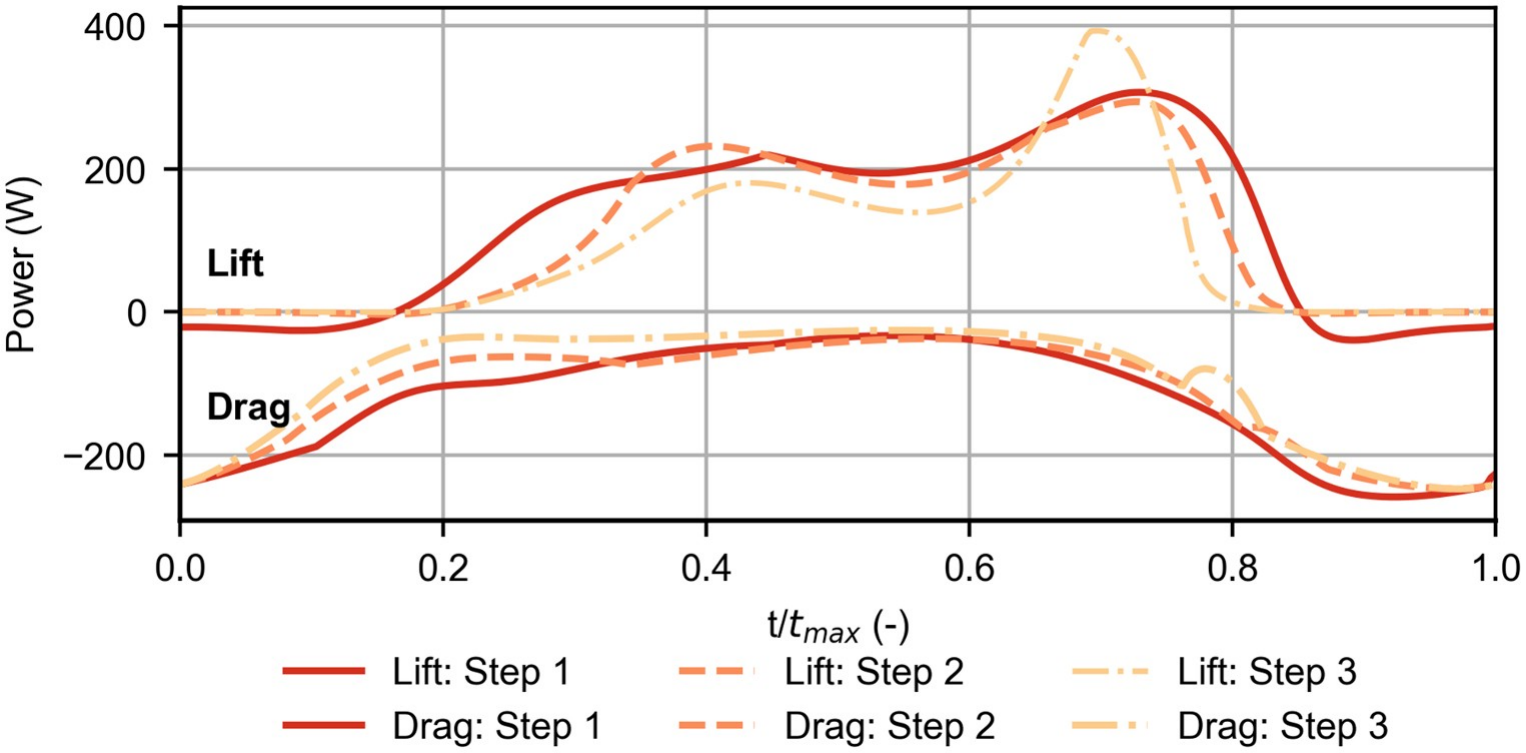
Effect of the transition height. Evolution of energy rates associated to lift and drag, for different transition heights of the step wind profile.

The step 3 wind profile requires higher wind speed since the vehicle is forced to climb for longer time without the presence of wind, i.e. without extracting energy (Fig 10). When it reaches the 15-meters altitude, its airspeed is very small and, as a consequence, there is a reduction in the lift available to provide energy, when compared with the other two cases. As a result, the only possibility to extract the same amount of energy is to have stronger wind.

The difference between step 1 and 2 (*b* = 5 and *b* = 10) is not as significant because lift depends on the square of the airspeed. So, as the airspeed decreases, the energy-extraction decreases with the square of the airspeed difference, which needs to be compensated with increasing wind speed. Since the trajectory for b = 15 (step 3) implies flying with lower airspeeds than for the other two cases, the impact of the transition height becomes more relevant. The evolution of energy gains and losses, for different steepness *k* of the wind profile (steps 1, 4 and 5), is almost negligible, hence is not reported in the paper.

### Effect of the initial conditions

All results presented until now considered the same set of initial conditions. From the previous discussion, it seems reasonable to assume that, by changing the initial conditions of the vehicle (Table 1), the efficiency attributed to each wind profile may change. If the conditions are not suitable, the vehicle cannot extract the highest amount of energy from the profile and the feasibility of the trajectory may be compromised.

**Fig 11:**
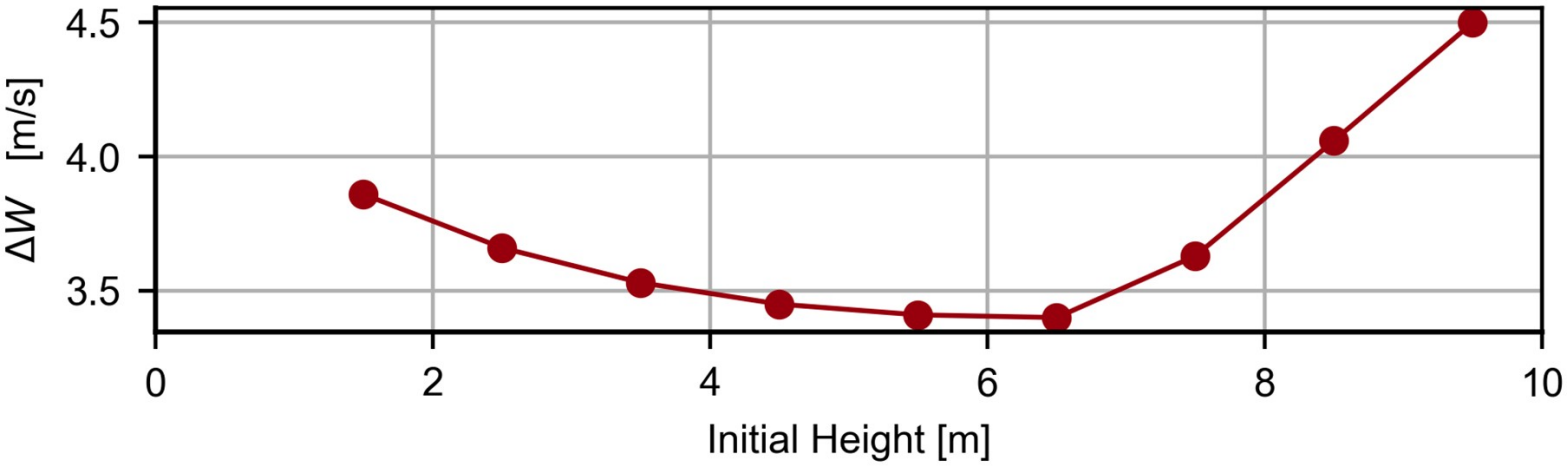
Effect of the initial height. Evolution of the required wind speed as a function of the initial height for a step-wind profile with b = 10 and k = 0.5.

Fig 11 presents the evolution of the required wind strength as function of the initial height for the case of the step 2 wind profile.

Fig 11 shows that there is an initial height that minimizes the required wind strength. The optimal altitude is around 6.5 meters. In fact, when the initial height is lower than the optimal value, the vehicle is forced to climb and descend without extracting energy for more time. On the contrary, if the initial height is larger than the optimal value, the lower turn will occur in the presence of higher wind speed, which increases the energy losses. It follows from Fig 11 that, among the two effects, the more negative contribution is given by the higher wind speed in the lower turn.

**Fig 12:**
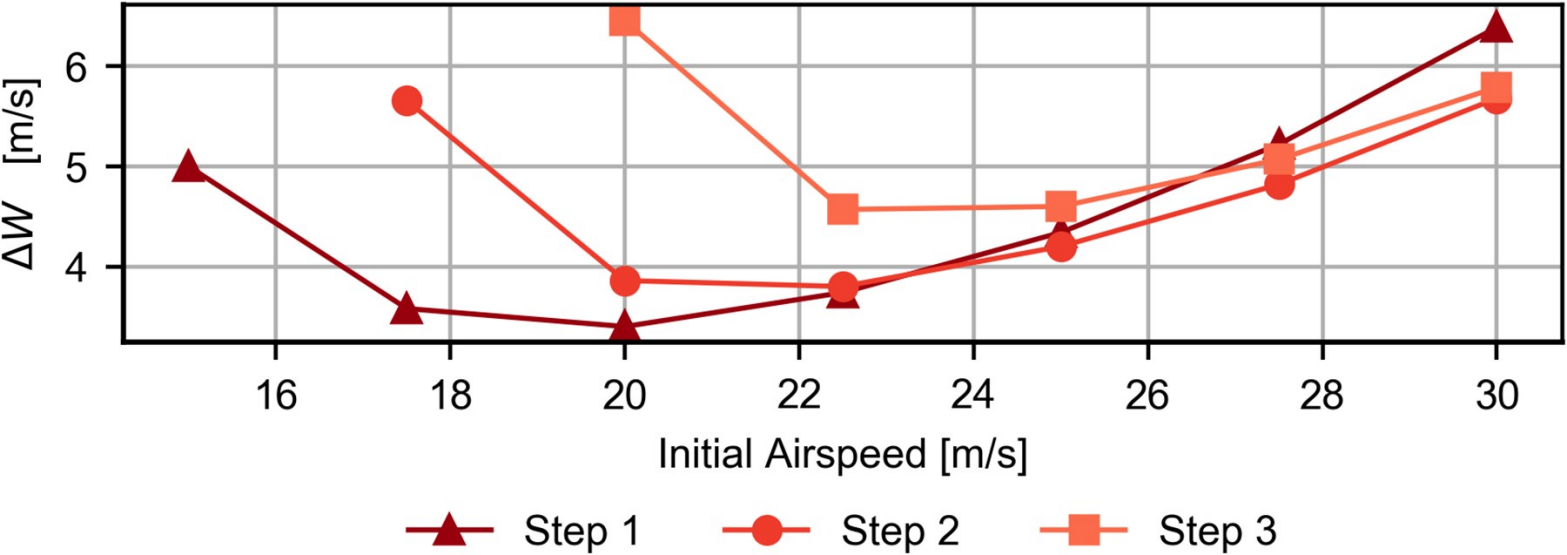
Effect of the initial airspeed. Evolution of the required wind strength as a function of the initial airspeed for three different step-wind profiles with b = 5, 10 and 15, and k = 0.5.

Fig 12 shows the evolution of the required wind speed difference, Δ*W*, as a function of the initial airspeed, for the cases of step 1, 2 and 3 (*b* = 5, 10 and 10, respectively), with k = 0.5.

From Fig 12 it follows an initial airspeed that minimizes the required Δ*W*. In addition, depending on the initial airspeed, the most efficient step-wind profile changes. As expected, the step with the lowest transition height is the most efficient profile for low airspeeds, while those with higher transition heights are better for higher initial airspeeds.

Some explanation is due for the above cases. At lower than optimal initial airspeeds, the increase of the required wind speed is the result of a decrease in the capacity of the vehicle to extract energy. The lift depends on the square of the airspeed so its contribution to the energy is reduced at lower airspeed. In addition, for step wind profiles with high transition heights, there are not feasible trajectories for low initial airspeeds since the vehicle is not able to reach the transition height.

At higher than optimal initial airspeeds, the increase of the minimum wind speed can be explained by two factors. On the one hand, although the energy extraction increases with the airspeed, so does the drag, demanding higher wind speeds. On the other hand, the limitation imposed by the maximum admissible load factor limits the maximum airspeed and turn rate which the vehicle can reach, resulting in less efficient trajectories.

### Effect of vehicle constraints

**Fig 13:**
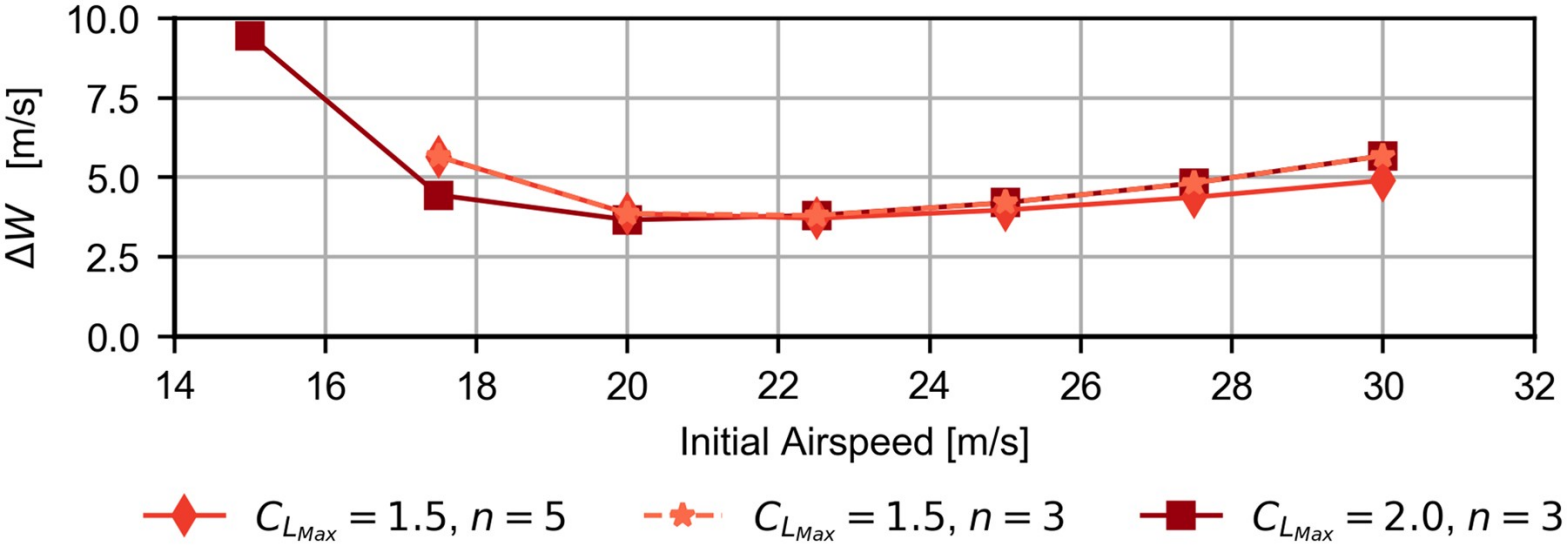
Effect of the vehicle constraints. Evolution of the required wind strength as a function of the initial airspeed for different vehicle constraints (lift coefficient *C*_*L*_ and load factor *n*).

From the previous discussion about the variation of performance with initial airspeed, it was found that the maximum lift coefficient and maximum load factor influence the feasibility of dynamic soaring maneuvers. Fig 13 presents the evolution of the required wind strength as a function of the initial airspeed for different vehicle constraints while considering the case of the step 2 wind profile (*b* = 10, *k* = 0.5).

The results related to $C_{L_{max}}=1.5,\;n=3$ (star symbol) are superposed to to the diamond symbols ($C_{L_{max}}=1.5,\;n=5$) for initial airspeeds less than 23 *m*/*s* and to the square symbols ($C_{L_{max}}=2,\;n=3$) for larger values.

At $C_{L_{max}}=1.5$, the minimum initial airspeed for a feasible Rayleigh cycle is independent on the maximum load factor. Increasing the maximum lift coefficient to $C_{L_{max}}=2$ makes it possible to reduce significantly the initial airspeed, but with a higher required Δ*W*.

It follows that the maximum lift coefficient and the maximum load factor change the feasibility region. For instance, for an initial airspeed of 15 m/s and a step wind profile with transition height at 10 meters, there is not a feasible trajectory when the maximum lift coefficient is 1.5. On the contrary, if the maximum lift coefficient is increased to 2, a feasible trajectory can be obtained.

### Trajectories for excess wind conditions

The trajectories considered up to now were obtained with the purpose of minimizing the necessary wind speed. Finally, it is interesting to analyze the case of wind strength higher than the minimum required.

The aim is to find trajectories that, by exploiting favorable wind conditions, can be used for surveillance missions. Thus, the obtained trajectories must be closed, single-loop and energy-neutral, in order to have motions with simple control schemes, and that are continuously repeatable in constant wind conditions. For these trajectories the two characteristics to be optimized are endurance and range.

**Fig 14:**
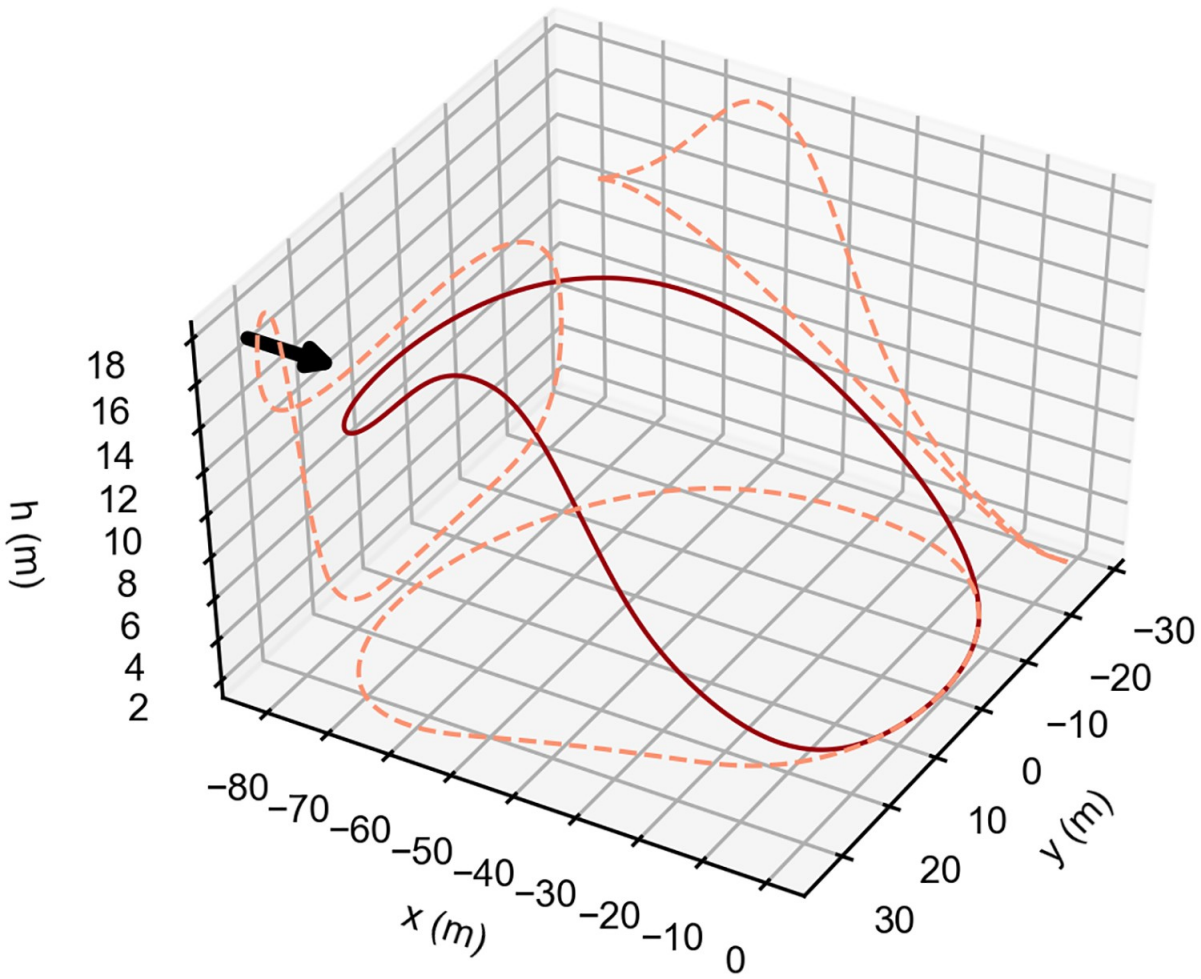
Possible surveillance trajectory. Optimal single-loop, energy-neutral trajectory that maximizes the time aloft. The arrow indicates the wind direction. The vehicle travels in the clockwise direction.

Fig 14 presents the result that maximizes the flight time of the closed, single-loop, energy-neutral trajectory for a step-wind profile with b = 5 and k = 0.5, and with a maximum wind strength (A of Eq (15)), equal to 5m/s.

Fig 14, shows the different behavior of the vehicle when compared with the previous cases (see Fig 8). The trajectory obtained is still a simple single-loop closed trajectory, but after the initial climb, there is a small descent into the wind, that generates a loss of energy. Afterwards, the upper turn occurs at almost constant height, followed by the final descent and by the lower turn.

The resulting trajectory can be explained by the fact that, to have an energy-neutral loop in excess wind conditions, it is necessary to dissipate the additional energy gained due to the stronger wind speed. Since the objective is to increase the flight time, the solution is to dissipate the excess energy in a way that the trajectory time increases. The small descent into the wind, combined with the longer upper turn, allows for such goal.

**Fig 15:**
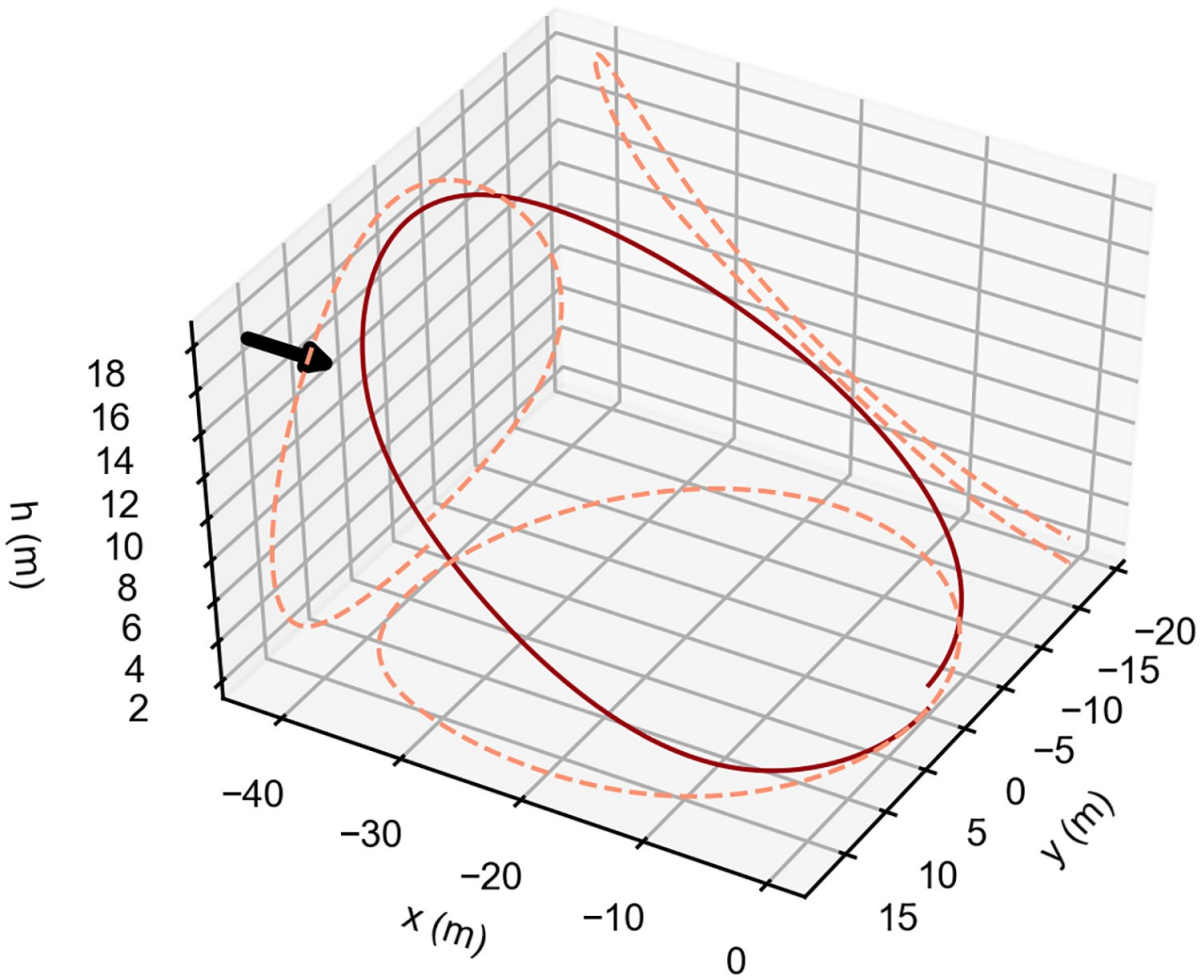
Other possible approaches. Example of dynamic soaring trajectory with altitude gain of 1 meter at the end of one loop and without loss of kinetic energy.

There are other alternatives to use excess wind conditions beyond those considered in this section, that bring to non-energy-neutral trajectories. For instance, it is possible to use the larger energy-extraction to increase the final airspeed or height of the vehicle, in each loop. In other words, in excess wind conditions, it is possible to climb (Fig 15) or accelerate without supply of energy.

## Concluding remarks

Since Leonardo da Vinci, the flight of birds, able to extract energy from the wind, was observed with admiration and described with unbelievable intuition and deepness for the scientific knowledge available at the time. After a long period of time, Lord Rayleigh analyzed the problem with rare brightness together with more proper scientific tools and proposed a model, very simple but capable of providing a clear explanation of the physical phenomena.

In the second half of the last century, along with the progress in aerodynamics and flight mechanics, many studies were conducted to interpret in mathematical terms the suggested model and to define the relevant equations for the search of the related trajectories. The growth of computational capabilities multiplied the results on the subject, through simulation and optimization approaches, that gave more and more contributions to the clarification of dynamic soaring either for birds or for autonomous vehicles inspired by the flight of birds. Concerning the equivalence of the two different reference frames to be adopted for the solution of the equations as well as the most suitable form of kinetic energy to be considered (i.e. based on ground speed or on airspeed), a full clarification has been finally obtained in recent years [12].

Currently, we thought reasonable to consider again the original model of Rayleigh for the assessment of its feasibility for various wind conditions and vehicle characteristics, but in the framework of smooth approximations of the step wind profile. The optimization procedure adopted to identify the trajectories has been described together with the most significant results for several type of objective functions and values of the control parameters. The relevant mechanisms of energy transfer along the phases of the Rayleigh cycle are discussed. The most significant variables for the energy harvesting process are the lift/drag ratio and the velocity profile of the wind. In fact, to perform dynamic soaring maneuvers, it is necessary to have a maximization of the energy extraction on the higher part of the trajectory and a minimization of the losses on the lower part. Also, both the initial height and airspeed were found to be very important conditions to minimize the required wind strength. In addition, the aerodynamic and structural limits of the vehicle, in the form of the maximum lift coefficient and load factor, influence the feasibility region and, in general, may limit the performance of the dynamic soaring trajectories.

The potential interest for application to the flight of autonomous vehicles, along closed neutral trajectories, for instance to surveillance purposes, strengthens the present investigation also for natural wind conditions less extreme with respect to the one considered here. Trajectories especially designed for surveillance missions are proposed and, in case of stronger wind conditions, it is possible to utilize the excess energy to extend the time aloft or the length of the trajectory.

## Appendix

### Method verification

The computational methodology was verified by comparing the results obtained with the results obtained by Sachs [10], in which a open dynamic soaring trajectory that minimizes the required wind strength in logarithmic wind conditions was presented, resulting in a required minimum wind strength of 8.6 m/s.

**Fig 16:**
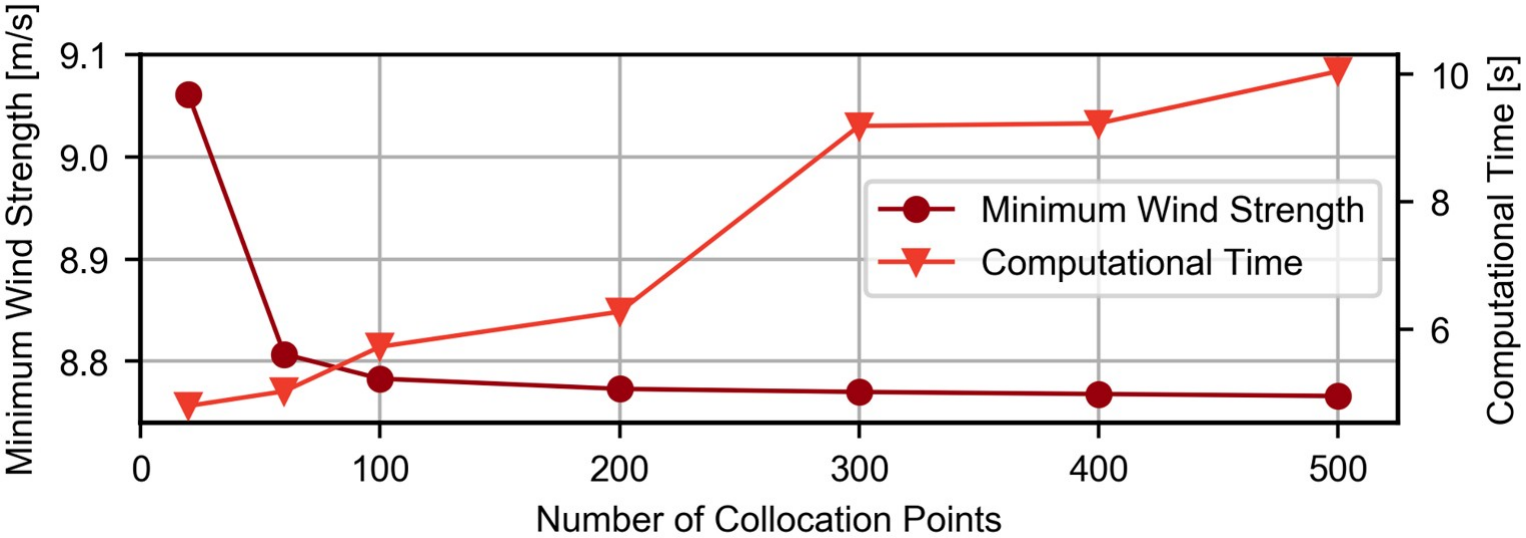
Convergence study. Accuracy and convergence study of the optimization procedure.

Fig 16 presents the convergence study of the number of collocation points for the optimization procedure. Looking at the data, it is possible to verify that the value has already converged to 8.77 m/s with N equal to 200, corresponding to a difference of 2% when compared with values obtained by Sachs [10]. The small discrepancy is most probably due to the fact that the exact conditions used by Sachs could not be recreated since not all required information is available regarding the optimization procedure. Nevertheless, it is safe to conclude that the results obtained using the trapezoidal rule are accurate when N is equal or greater than 200.
